# Molecular and Cellular Characterization of the Tomato Pollen Profilin, *LePro1*

**DOI:** 10.1371/journal.pone.0086505

**Published:** 2014-01-21

**Authors:** Long-Xi Yu, Mandayam V. Parthasarathy

**Affiliations:** Department of Plant Biology, Cornell University, Ithaca, New York, United States of America; Institute of Botany, Chinese Academy of Sciences, China

## Abstract

Profilin is an actin-binding protein involved in the dynamic turnover and restructuring of the actin cytoskeleton in all eukaryotic cells. We previously cloned a profilin gene, designated as *LePro1* from tomato pollen. To understand its biological role, in the present study, we investigated the temporal and spatial expression of *LePro1* during pollen development and found that the transcript was only detected at late stages during microsporogenesis and pollen maturation. Using antisense RNA, we successfully knocked down the expression of *LePro1* in tomato plants using stable transformation, and obtained two antisense lines, A2 and A3 showing significant down-regulation of *LePro1* in pollen resulting in poor pollen germination and abnormal pollen tube growth. A disorganized F-actin distribution was observed in the antisense pollen. Down-regulation of *LePro1* also appeared to affect hydration of pollen deposited on the stigma and arrested pollen tube elongation in the style, thereby affecting fertilization. Our results suggest that *LePro1* in conjunction with perhaps other cytoskeletal proteins, plays a regulatory role in the proper organization of F-actin in tomato pollen tubes through promoting actin assembly. Down-regulation of *LePro1* leads to interruption of actin assembly and disorganization of the actin cytoskeleton thus arresting pollen tube growth. Based on the present and previous studies, it is likely that a single transcript of profilin gives rise to multiple forms displaying multifunctionality in tomato pollen.

## Introduction

Actin and actin-binding proteins (ABPs) are fundamental elements of the cytoskeleton which together play an important role in plant cell morphogenesis, mitogenesis, mobility and other cellular processes [1], [2], [3], [4], [5]. The actin cytoskeleton is composed of a network of actin filaments whose precise organization is regulated by a number of actin binding proteins. One of them is profilin, a small (12–15 kDa) monomeric actin binding protein. The functions associated with the action of profilin, which may be temporally and spatially correlated, include: (1) actin monomer and filament end binding [6], [7]; (2) positive or negative control of actin nucleation and polymerization [8], [9], [10], [11]; (3) participation in the phosphoinositide secondary messenger signaling pathway [4], [12], [13], [14]; (4) poly-L-proline binding to target profilin-actin monomer complexes to sites of actin assembly [15].

In plants, profilin was first identified as a ubiquitous allergen from birch pollen [16]. Later, cDNA clones encoding profilin were isolated from other species such as maize, timothy grass, wheat, tobacco, common bean and Arabidopsis [17]. Functional assessments of plant profilins have been carried out in several species. Ramachandran et al. [18] analyzed *in vivo* functions of Arabidopsis profilin by generating transgenic plants carrying a 35S-PFN-1 or 35S-antisense PFN-1 transgene. Their results indicated that Arabidopsis profilins play a role in cell elongation, cell shape maintenance, polarized growth of root hairs, and in determination of flowering time. In maize, class I profilins inhibited hydrolysis of phosphatidylinositol-4,5-bisphospholipase more strongly than did class II profilin. In contrast, class II profilins had higher affinity for poly-L-proline and sequestered more monomeric actin than did class I [19]. In Arabidopsis, five profilin isoforms have been isolated. They are distinctively regulated by development and may play distinctive roles [20]. Vidali et al. [21] used a transient RNA interference approach to knockdown profilin expression in the *Physcomitrella patens* and demonstrated that the F-actin was disorganized and the tip growth was inhibited in the profilin-defective moss cells. More recently, multifunctionality of pollen profilin isovariants has been characterized using sequence comparison in several plant species. It has been suggested that profilin multifunctionality might be due to natural variation through its isovariants [22].

We previously cloned a pollen profilin gene from tomato pollen, *LePro1* [23], and found evidence suggesting that *LePro1* is a pollen-specific profilin [23]. To investigate the biological role of *LePro1*, in the present study, we further characterized this gene by sequence alignment, protein structure, genomic organization, *in situ* hybridization, antisense RNA to knock-down the gene expression in transgenic plants, and undertook sequence comparison and gene structure analysis.

## Materials and Methods

### Plant Materials

Tomato (*Solanum lycopersicum*) cv MoneyMaker, was used in this study and grown in the Cornell greenhouse under normal conditions until pollen maturation.

### DNA and RNA Gel Blots and *In situ* Hybridization

Genomic DNA was extracted from young leaves of tomato plants according to Fulton et al. [24]. Total RNA was extracted from mature pollen as previously described [23]. For DNA and RNA gel blot, ^32^P-labeled *LePro1* cDNA probe was hybridized to the immobilized Hybond N membrane (Amersham) containing DNA or RNA, respectively according to Sambrook et al. [25]. For *in situ* hybridization, tomato flower buds of 3, 6, 9, 12 and 15 mm in length, representing different development stages, were collected and fixed immediately in 3∶1 ethanol:acetic acid fixative, followed by dehydration, embedding, sectioning and hybridization processes as previously described [23]. Single-strand sense and antisense RNA were synthesized by *in vitro* transcription of *LePro1* cDNA cloned in pCRII (Invitrogen). They were then labeled with digoxigenin (DIG) using the RNA Labeling Kit (Boehringer Mannheim) and *in situ* hybridized to tomato sections as described previously [23].

### Vector Construction and Transformation

Sense and antisense constructs were made by insertion of *LePro1* cDNA into the promoter less binary vector pBI101 (Clontech) in sense and antisense orientations respectively. The LAT52 promoter and the NOS terminator were used for controlling expression. Both sense (pB-Lat-LePro1S) and antisense (pB-Lat-LePro1A) constructs were then introduced into tomato plants using *Agrobacterium* transformation according to Fillatti et al. [26] and Frary and Earle [27]. Briefly, sterile cotyledon and hypocotyl explants were cultured on a feeder layer containing tobacco NT1 suspension cells, followed by co-culturing the explants with the strain LBA4404 harboring the sense or antisense constructs for 48 h. The explants were then transferred to selective regeneration medium containing 50 mg/l kanamycin and 100 mg/l timentin and subcultured every 3 weeks until green shoots formed. Shoots about 2 cm tall were excised and transferred to rooting medium containing the same antibiotics. Rooted plants were then transplanted to soil and grown in a humid growth chamber and finally transferred to the green house. Positive transgenic plants were determined by PCR amplification of genomic DNA using gene-specific primers according to Li et al. [28].

### Protein Extraction and Immunoblotting

Mature pollen grains were collected and immediately frozen in liquid nitrogen. Total soluble proteins were extracted according to Darnowski [29]. Protein concentration was determined using the BioRad protein assay kit (BioRad, CA). Ten micrograms of proteins per sample were loaded onto 14% SDS-polyacrylamide gel and separated by electrophoresis. Proteins were transferred to Hybond ECL membranes (Amersham) by trans-blot cell (BioRad). The membranes were then incubated with polyclonal anti-tomato profilin antibody [29] and monoclonal anti-pea actin antibody [30] for one hour. Color was developed using 4-chloro-1-naphathol and hydrogen peroxide according to Bollag and Edelstein [31]. Protein signals were scanned with Fluor-S™ multiImager (Bio-Rad) and quantified by the unit density of pixels using the enclosed Quantity 1 software.

### Pollen Germination and Morphological Analysis

For *in vitro* pollen germination, pollen grains were collected as described previously [23] and suspended in tomato pollen germination medium (TPGM) containing 10% sucrose, 0.01% KNO_3_, 0.01% H_3_BO_3_, 0.02% MgSO_4_, 0.06% Ca(NO_3_)_2_ and 20 mM MES (pH 7.0). They were then spread on the TPGM solidified with 0.5% agarose in 7 cm diameter petri dishes and germinated at room temperature in the dark for 12 hrs. Pollen germination was examined by a Zeiss Axiovert inverted microscope. For *in vivo* pollen germination, flowers subject to pollination were labeled and anthers were carefully removed one day before anthesis. They were covered with plastic bags to prevent cross contamination. The following morning, fresh pollen grains were collected from the donor plants and placed on the stigmas of the receptor plants using a painting brush. The bags were placed on the flowers immediately after pollination. After 24 h, the pollinated pistils were removed from the plants and fixed immediately in 3∶1 ethanol:acetic acid 1 h at room temperature, the pistils were then transferred to 8 M NaOH for softening overnight, followed by washing 3 times in distilled water and incubating in 0.1% aniline blue (w/v in 0.1 M H_3_PO_4_) overnight in the dark. The stained pistils were destained in distilled water for 1–3 h and then squashed between a cover slip and a slide in the presence of a drop of glycerol. The whole-mounted pistils were then examined by a Zeiss Axioplan 2 microscope with an AMCA filter. For electronic microscopy, dry and hydrated pollen grains were examined using a Hitachi 4500 Field Emission Scanning Electron Microscope (FESEM). For low temperature scanning electron microscopy (LTSEM) of frozen-hydrated specimens, pistils containing pollen that were deposited the previous day on stigmas were removed from the flowers, and frozen in liquid nitrogen in the following morning. They were then viewed by the same FESEM equipped with a BALTEC cryo-stage.

### Actin-staining and F-actin Quantification

Actin staining was carried out according to Gibbon et al. [32] with minor modification. Briefly, germinated pollen grains were fixed in the fixative containing 1 volume of TPGM plus 4% paraformaldehyde, 4% sucrose and 0.6 mM 3-maleimidobenzoic acid N-hydroxysuccinimide ester (MBS) for 30 min. The pollen grains were rinsed three times in TPGM plus 0.05% Nonidet P-40 and gradually changed into TBS-Tween (50 mM Tris, 200 mM NaCl, 0.05% Tween 20, 300 mM sucrose and 5 mM DTT, pH 7.4). They were then stained with 0.001 mM rhodamine phalloidin for 1 hour at room temperature in the dark, washed once with TBS-Tween without DTT. The stained pollen grains were mounted in the same buffer, and fluorescence images were taken using a Leica DMRE2 laser confocal microscope. F-actin was quantified by measuring phalloidin binding sites in pollen stained as described above, followed by washing three times in TPGM plus 0.05% Nonidet P-40. F-actin levels were determined by eluting bound phalloidin from cells into methanol and measured by a spectrofluorometer. Fluorescence values were converted to phalloidin value per pollen gain/tube. The average diameter of 12 µm was used for calculating the volume of pollen grains. For pollen tube, the average length and width were used to calculate the volume of a cylinder. The quantification of total actin was done by the Enzyme-linked immunosorbent assay (ELISA). The same anti-actin antibody used in the western blot was also used for actin quantification.

### Data Analysis

Experimental data was statistically analyzed using ANOVA. Standard errors and P values were presented in the corresponding figure legends.

## Results

### Genomic Organization of *LePro1*

**Figure 1:**
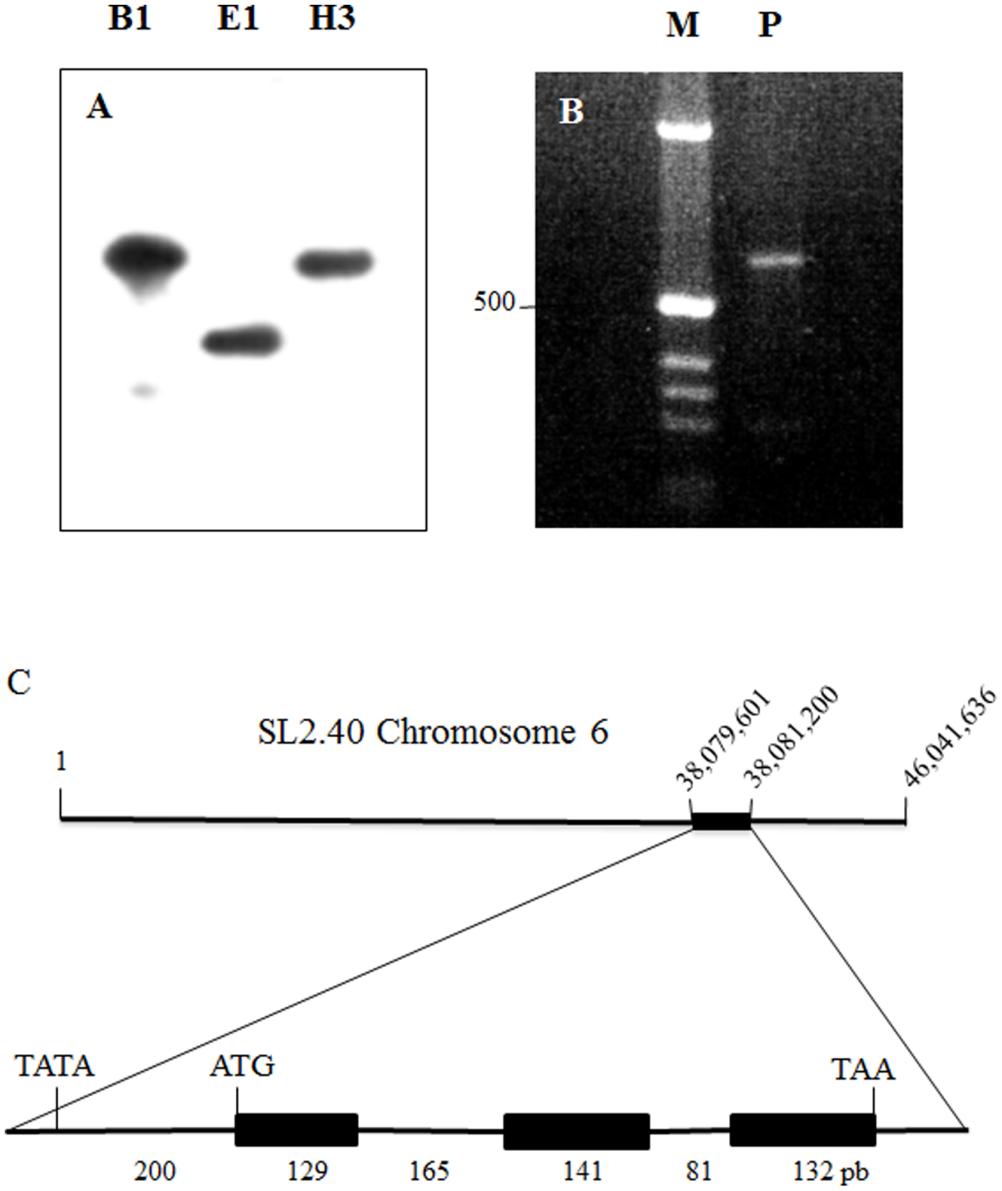
Genomic organization of *LePro1*. Panel **A**, DNA gel blot, showing single hybridization signal in each digestion by BamH1 (B1), EcoR1 (E1) and Hind III (H3). Panel **B**. genomic DNA fragment was amplified by polymerase chain reaction using 5′- and 3′- primers derived from the *LePro1* coding sequence. A ∼650 bp PCR product (P) was obtained. Left column in panel b shows DNA size markers (M). Panel **C**, top row shows chromosome location of *LePro1* based on the genome sequencing database of the Sol Genomics Network (http://solgenomics.net). Bottom row shows the structure of *LePro1* containing 3 exons and 2 introns with 648 bp in length from the start codon (ATG) to the stop codon (TAA). A TATA box was found at 200 bp upstream to the start codon.

To investigate the genomic organization of *LePro1,* we carried out DNA gel blot analysis. ^32^P-labeled *LePro1* cDNA probe was hybridized to tomato genomic DNA digested by BamH I, EcoR I and Hind III restriction enzymes, respectively. A single band was found in all three digestions (Fig. 1a), suggesting that *LePro1* is likely encoded by a single copy gene. To confirm this result, we synthesized a pair of primers derived from the 5′ and 3′ of the coding region of *LePro1* cDNA and amplified tomato genomic DNA by polymerase chain reaction (PCR). Again, a single band was obtained by gel electrophoresis of the PCR product (Fig. 1b). This DNA product is about 650 bp. This size is about 200 bp larger than the cDNA coding region, suggesting that there is an intron between the two primers. To confirm this result, we performed a BLAST search in the tomato genomics sequencing database (http://solgenomics.net) using *LePro1* cDNA sequence as a query. We found a homolog genomic sequence highly similar (99%) to *LePro1* in the region between 38,079,001 bp and 38,081,200 bp on chromosome 6 (Fig. 1C, top). The alignment of *LePro1* against the tomato genomic sequence revealed 3 exons and 2 introns with 648 bp in length from the start codon (ATG) to the stop codon (TAA) (Fig. 1C bottom). This is very close to the length of the PCR product mentioned above (Fig. 1B), indicating that the PCR indeed amplified the genomic sequence for *LePro1*. There is a TATA box at the 200 bp upstream to the start codon (Fig. 1C, bottom), representing a key element of the promoter region.

### Temporal and Spatial Expression of *LePro1* during Pollen Development

**Figure 2:**
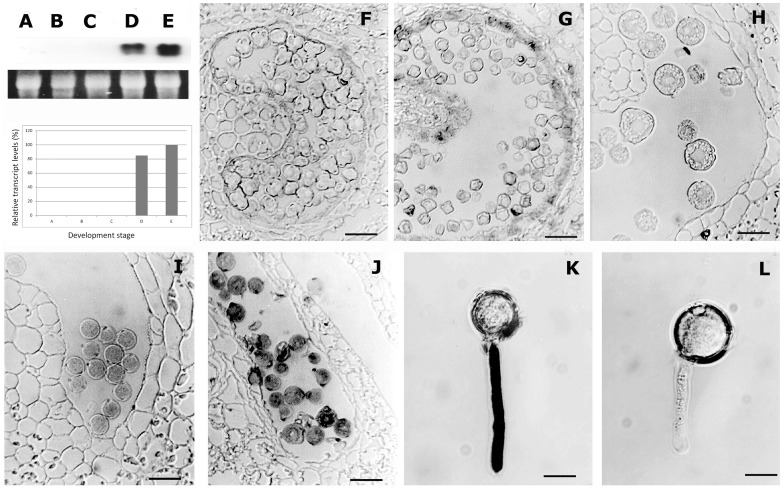
RNA gel blot and *in situ* hybridization of *LePro1* in developing tomato flowers. The panel on the tope left shows RNA gel blot analysis with hybridization signals on the top and loading control on the bottom. **A, B, C, D** and **E** represent anther sections from 3, 6, 9, 12 and 15 mm length flower buds, respectively. **F, G, H, I** and **J** are images showing *in situ* hybridization of DIG- labeled antisense *LePro*1 to tomato anther sections from flower buds at the same developmental stages as shown in **A, B, C, D** and **E,** respectively. **K** shows germinated pollen hybridized with antisense *LePro1* probe, and **L** represents germinated pollen hybridized with sense *LePro1* probe as control (Scale bar = 40 µm in **F–J** and 20 µm in **K** and **L**).

To analyze the expression pattern of *LePro1* during microsporogenesis, we first carried out RNA gel blot analysis (Fig. 2 A–E) in a series developmental stages of flowers. We used flower bud length for determining development stages. Hybridization signals were found in pollen of 12 and 15 mm flower buds (Fig. 2, D and E). The transcripts became abundant as pollen maturing (Fig. 2, plot). The highest level of transcript was found in pollen of 15 mm flower buds (Fig. 2, E and plot), which is about 1–2 days before anthesis. To confirm this result, we then performed *in situ* hybridization using the similar series of developing flowers (Fig. 2, F-K). In this experiment, the transcript of *LePro1* was not detected until the tetrad stage of pollen development and stronger signals were detected in the mature pollen. *LePro1* transcripts become more abundant as pollen germinates (Fig. 2, J and K). The result of *in situ* hybridization was consistent with that of the gel blot. Both suggested that *LePro1* is temporally and spatially expressed late during pollen development and maturation.

### Characterization of Tomato Transgenic Plants Carrying Antisense and Sense *LePro1*

**Figure 3:**
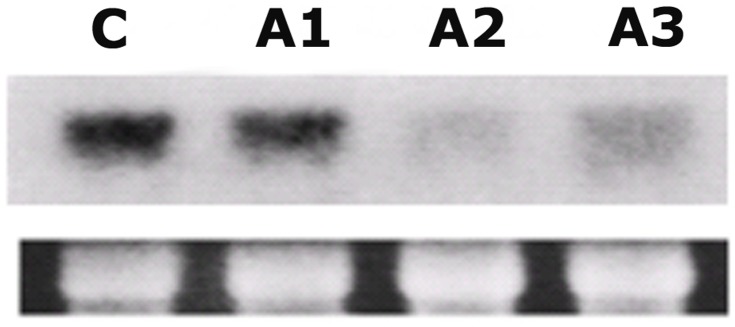
RNA gel blot analysis of the *Lepro1* transcript in pollen grains of wildtype (C) and antisense plants (A1–A3), probed with ^32^P-labeled *LePro1* cDNA. The bottom row shows ribosome RNA (rRNA) stained with ethidium bromide as loading control.

**Figure 4:**
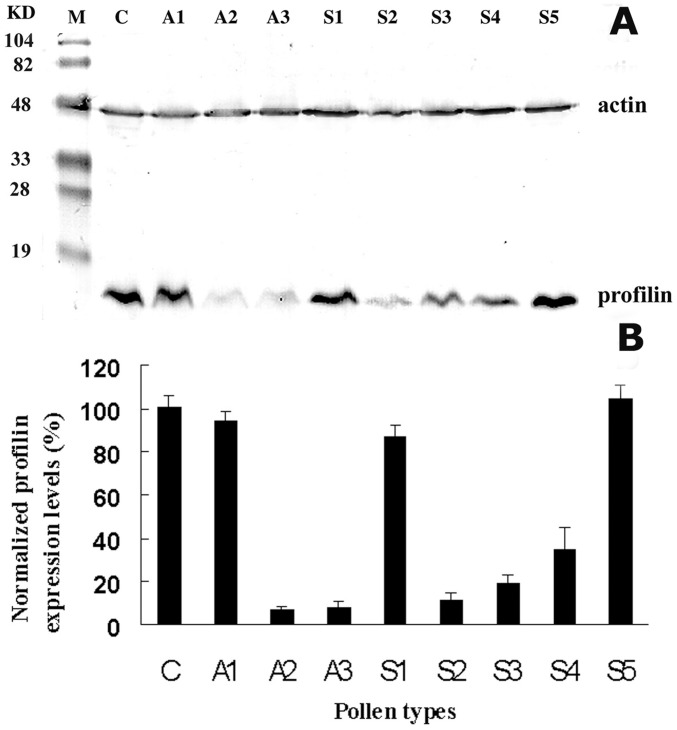
Immunoblot analysis of total soluble proteins extracted from pollen grains of wildtype (C), antisense (A1–A3) and sense (S1–5) plants. Ten micrograms of proteins per sample were loaded in each well. A mixture of two antibodies, monoclonal anti-actin antibody 3H11 and polyclonal anti-profilin antibody Tp1 were used. **A**, actin (43 kD) and profilin (14 kD) were detected within the same blot. The upper bands represent actin signals and the lower bands represent profilin signals. The left column shows molecular weight standards (M) in kD. **B**, protein signal intensities obtained with a Bio-Rad Fluor-S MultiImager densitometer.

As described in Materials and Methods, antisense and sense *LePro1* constructs were made and transformed to a tomato variety (MoneyMaker) using *Agrobacterium* transformation. Putative transgenic plants were regenerated and analyzed by PCR for transgene integration. Three antisense (A1–A3) and five sense (S1–S5) lines were obtained. Total RNA was extracted from pollen of transgenic and control plants and analyzed by RNA gel blot (Fig. 3). RNA level of *LePro1* was dramatically decreased in A2 and A3 lines (Fig. 3, A2 and A3), compared to wildtype control (C). While the transcript was not decreased in A1 pollen (Fig. 3, A1), suggesting no antisense effect occurred in A1 pollen. To confirm this finding, we also compared the protein level of *LePro1* in the lines by western blot using two antibodies. A tomato profilin-antibody was used for detecting the profilin and an actin-antibody was used as an internal control for actin expression. As shown in Fig. 4A, two bands were obtained for each line. The upper band represents the actin signal (43 kD) and the lower band represents the profilin signal (14 kD). Pollen from A2 and A3 antisense lines showed a significant reduction of the protein confirming the down-regulation of *LePro1* in A2 and A3. Whereas a normal level of profilin expression was found in A1 pollen, indicating no antisense effect in A1 line (Fig. 4A and B, A1)). In sense lines, S1 and S5 showed relatively higher levels of the profilin expression compared to that in nontransformed pollen, indicating an overexpression occurred in these lines. Lower levels of profilin expression were also found in the sense line S2, S3 and S4, indicating a possible co-suppression (Fig. 4A and B). As expected, the actin levels were comparable in all lines (Fig. 4A, upper bands).

**Figure 5:**
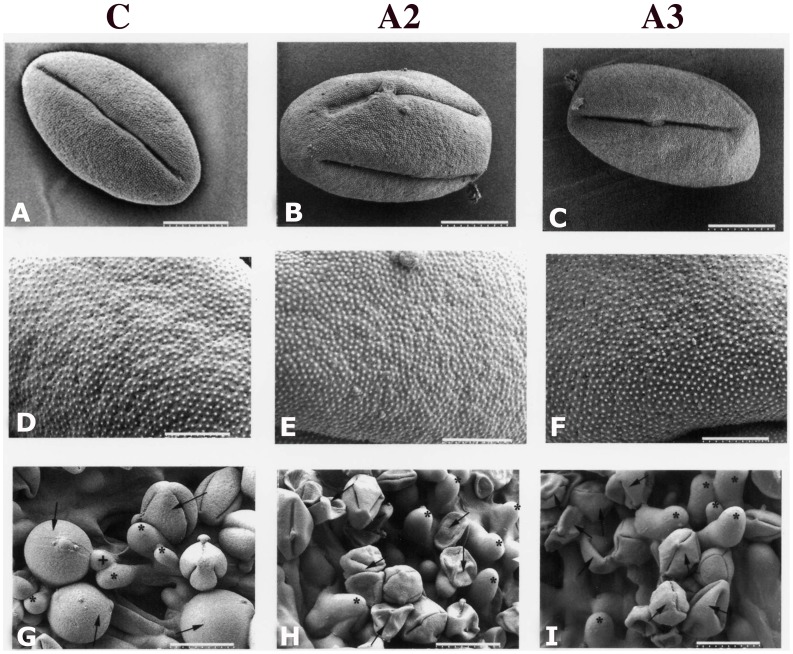
Ambient temperature SEM and low temperature SEM images of pollen in wildtype (panel C) and antisense (panels A2 & A3) plants. **A–C**, ambient temperature low magnification of images of dehydrated pollen grains showing no significant difference in size or morphology (Scale bar = 9 µm). **D–F**, ambient temperature higher magnification images of the dehydrated pollen grains from all three lines showing no significant difference in the exine microstructure (Scale bar = 3 µm). **G–I**, low temperature SEM of selfed pollen, 2 hours after pollination. **G**, most wildtype pollen grains deposited on stigma are fully or partially hydrated; **H** and **I**, most pollen grains from antisense plants are not hydrated. **G,** arrow indicates pollen in hydrated condition or **H** and **I**, in non-hydrated condition. **G,** asterisk indicates stigmatic cell, and+indicates emerging pollen tube (Scale bar = 23 µm in **G**, and 30 µm in **H** and **I**).

**Figure 6:**
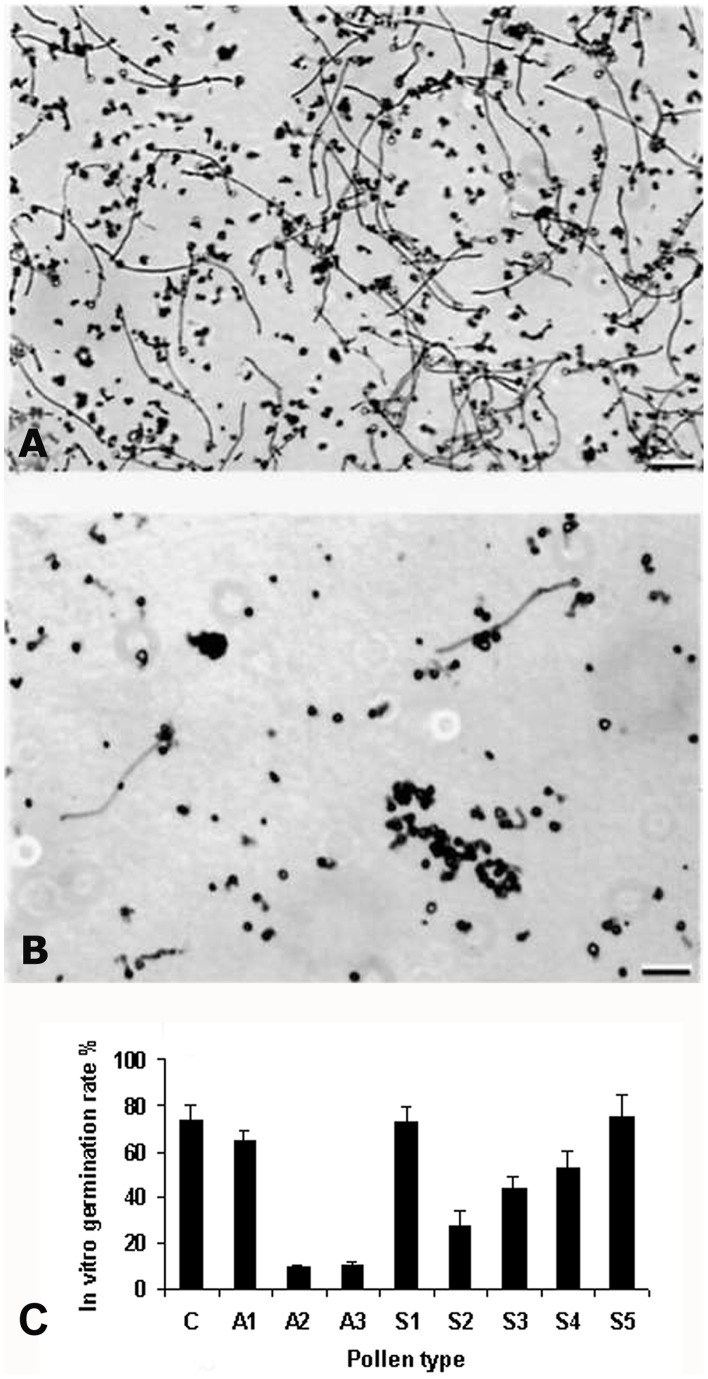
Images of *in vitro* germinated wildtype and antisense pollen. **A**, a high rate of pollen germination occurs in wildtype plants. **B**, a very low rate of pollen germination occurs in A3 line A few germinated pollen have short or abnormal pollen tubes (Scale bar = 100 µm). **C**, in vitro germination rate of different pollen type from antisense (A1–A3), sense (S1–S5) and wildtype plants (C). Error bars represent standard errors of 3 replications (p<0.001).

**Figure 7:**
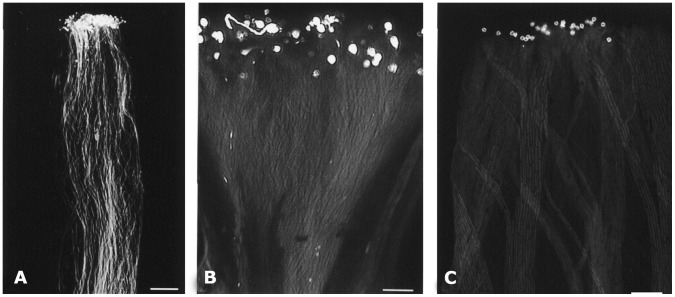
Comparison of *in vivo* germination of wildtype and antisense pollen. **A**, wildtype plants pollen showing normal pollen growth down through the style (Scale bar = 300 µm). **B**, low pollen germination and growth are seen when A3 pollen is deposited on the A3 stigma (selfing) (Scale bar = 80 µm). **C**, low pollen growth seen when A3 pollen is deposited on the wildtype plants stigma (crossing) (Scale bar = 160 µm).

To determine if there was any morphological change in antisense pollen, we conducted a scanning electron microscopy (SEM) analysis of pollen from wildtype and transgenic plants. The SEM did not reveal any significant difference in size, shape or exine architecture (Fig. 5, lane A–F). However, differences of pollen hydration between the wildtype and antisense lines were observed under LTSEM in which we conducted a comparative study of antisense and wildtype pollen deposited on stigma. Observations of such pollen using LTSEM indicated that compared to those from the wildtype plant, A2 and A3 lines had a larger number of pollen grains that failed to hydrate (Fig. 5, G-I). Since hydration is a critical step for pollen germination on the stigma, its failure may also lead to failure of pollen germination. In fact, the above results of pollen hydration correlate well with *in vitro* pollen germination (Fig. 6). Among pollen types analyzed, A2 and A3 lines showed the lowest germination rates, while A1, S1, S5 and wildtype (C) showed the highest rate. S2, S3 and S4 showed intermediate rates (Fig. 6). In addition to low pollen germination rates in A2 and A3 lines, the lengths of the pollen tubes were much shorter than those of the wildtype (Fig. 6A and B). *In vivo* germination also indicated a significant failure of pollen germination in the A3 line (Fig. 7). To confirm that the antisense effect in A3 is from pollen, not from the stigma, we performed cross-pollination between the antisense lines and the wildtype plants. Antisense pollen was placed on the wildtype stigma or the reverse, followed by examination of the pollinated pistils 24 hours after pollination. The result showed that selfing in wildtype plants showed a high rate of pollen germination (Fig. 7A) whereas A3 selfing showed a lower germination rate and poor tube growth (Fig. 7B). When A3 pollen was deposited on the wildtype plants stigma, the germination rate was much lower compared to selfing in wildtype plants (Fig. 7C). Results of A2 line crosses were similar to those of A3 lines (data not shown). These results indicated that the poor germination and tube growth in antisense pollen were indeed due to the antisense effect of *LePro*1 originating in the pollen, not in the stigma.

**Figure 8:**
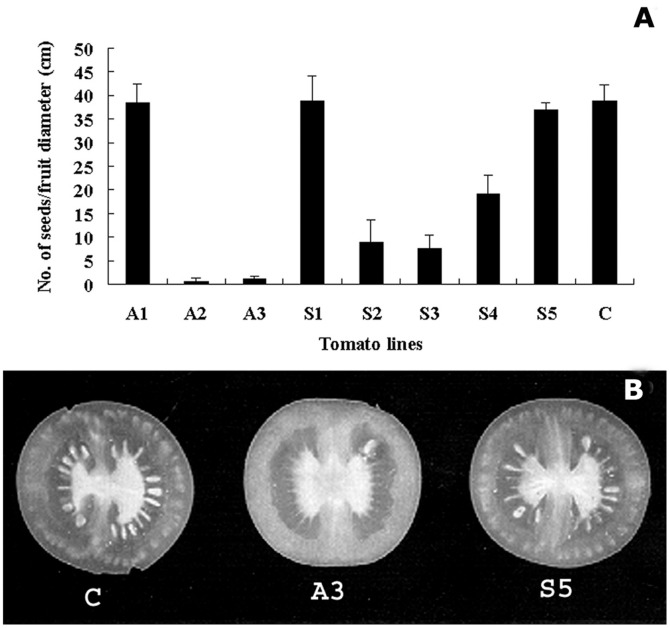
Comparison of seed-setting among *LePro1* sense, antisense and wild type plants Seeds were collected from mature fruits and counted. The number of seeds per cm fruit diameter was calculated (**A**). Error bars represent standard errors of 3 replications (p<0.001). **B**, shows examples of fruit sections from wild type (C), antisense 3 (A3) and sense 5 (S5) plants.

Poor pollen germination and tube growth would affect fertilization, resulting in poor seed-setting. To determine seed-setting among transgenic plants, we analyzed seed formation by calculating the number of seeds per cm fruit diameter (cmD) among antisense, sense and wildtype plants. We found that an average of 35 seeds/cmD was obtained in wildtype plants, while only 3 seeds/cmD were obtained in A2 and A3 lines (Fig. 8A). Among sense lines, S2, S3 and S4 showed 2–5 fold less seeds than that in wildtype plants. S1 and S5 lines had similar seeds number to those in wildtype plants. Examples of seed-setting in fruits from wildtype plants, A3 and S5 lines are illustrated in Fig. 8B.

### Actin Dynamics and F-actin Quantification

**Figure 9:**
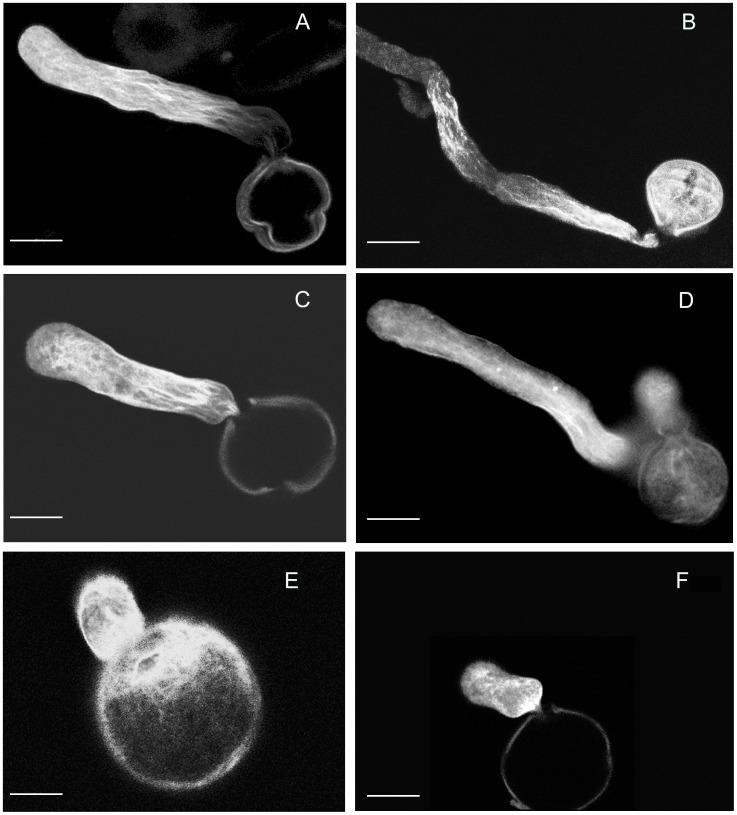
Confocal microscopy images of F-actin in germinated pollen in the wildtype and antisense line. Pollen and pollen tubes were stained with rhodamine phalloidin. A normal F-actin strand distribution was seen in wildtype pollen tubes (**A, B** and **C**). F-actin appears disorganized and pollen tube growth arrested in antisense pollen (**D, E** and **F**). Scale bars are: 10 µm in **A, B, C, D, F,** and 5 µm in **E**.

**Figure 10:**
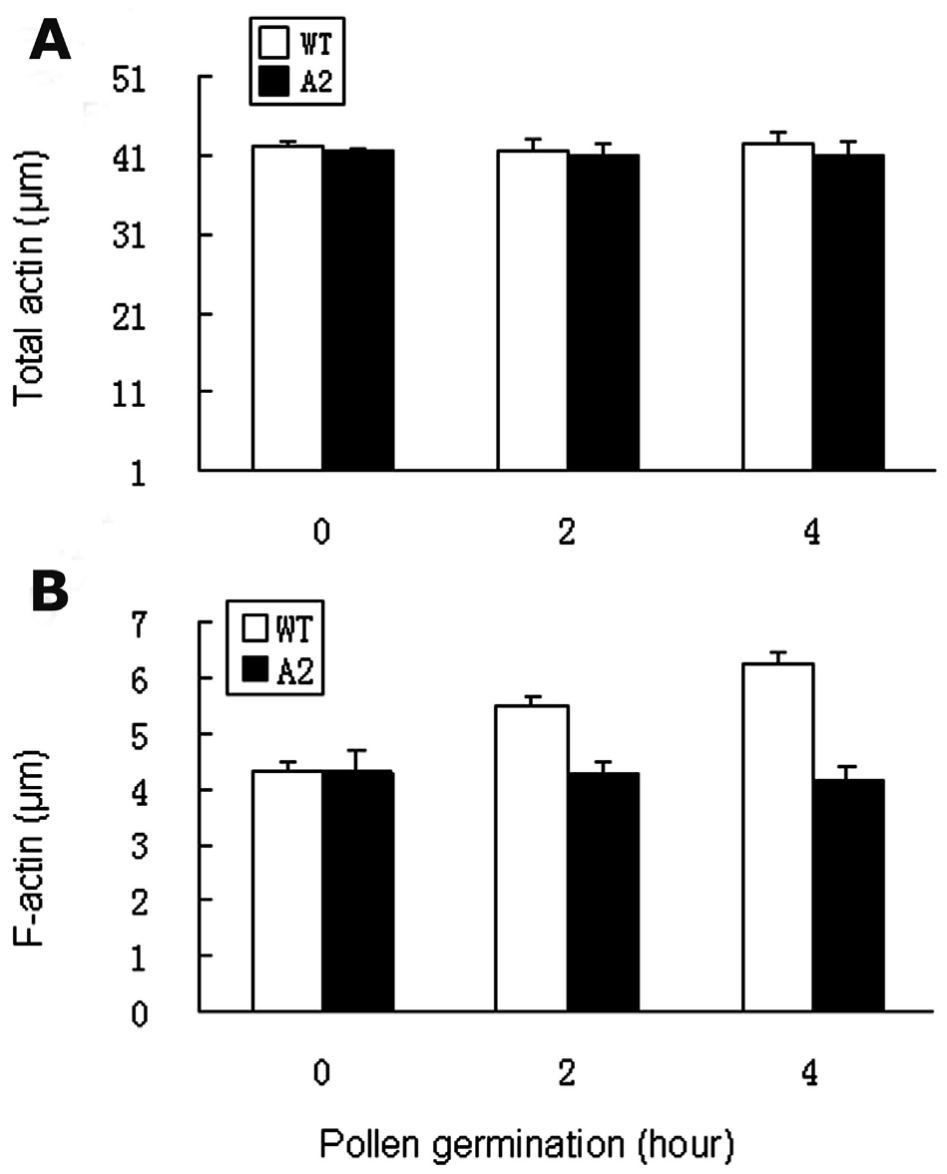
Quantification of total actin and F-actin in antisense (A2) and wildtype (WT) pollen. **A**, total actin quantified using anti-actin antibody followed by ELISA at the time course of 0, 2 and 4 hour germination. **B,** F-actin quantified using rhodamine phalloidin according to Gibbon and Staiger (2000). Error bars represent standard errors of 4 replications (p<0.005).

To analyze actin dynamics, we stained F-actin in wildtype and antisense pollen using rhodamine phalloidin and examined the F-actin organization in pollen tubes using confocal microscopy. Confocal images were taken from germinated pollen of wildtype and antisense lines (Fig. 9). A normal distribution of F-actin was observed in the wildtype pollen tubes (Fig. 9 A, B and C). However, due to the low germination rate (<10%) and abnormal tube growth, we only got a few phenotypes showing F-actin organization in the germinated antisense pollen (Fig. 9, D, E and F). Among those germinated, F-actin appeared at the beginning of pollen germination but disrupted after germination. Only a few showed tube elongation. Among those with elongated pollen tubes, F-actin bundles were detected in the basal portion of the pollen tubes but not in the apical or subapical regions (Fig. 9D). No F-actin cable was detected in the pollen tubes that stopped growing (Fig. 9E and F). To gain more information, we quantified total and F-actin levels in pollen and pollen tubes in antisense and wildtype lines during a time course of 0, 2 and 4 h germination. No significant difference was found at the total actin level between antisense and wildtype (Fig. 10A). However, the F-actin level increased as germination progressed in the wildtype, but not in the antisense pollen (Fig. 10B).

### Sequence Comparison and Structural Analysis of Plant Profilins

**Figure 11:**
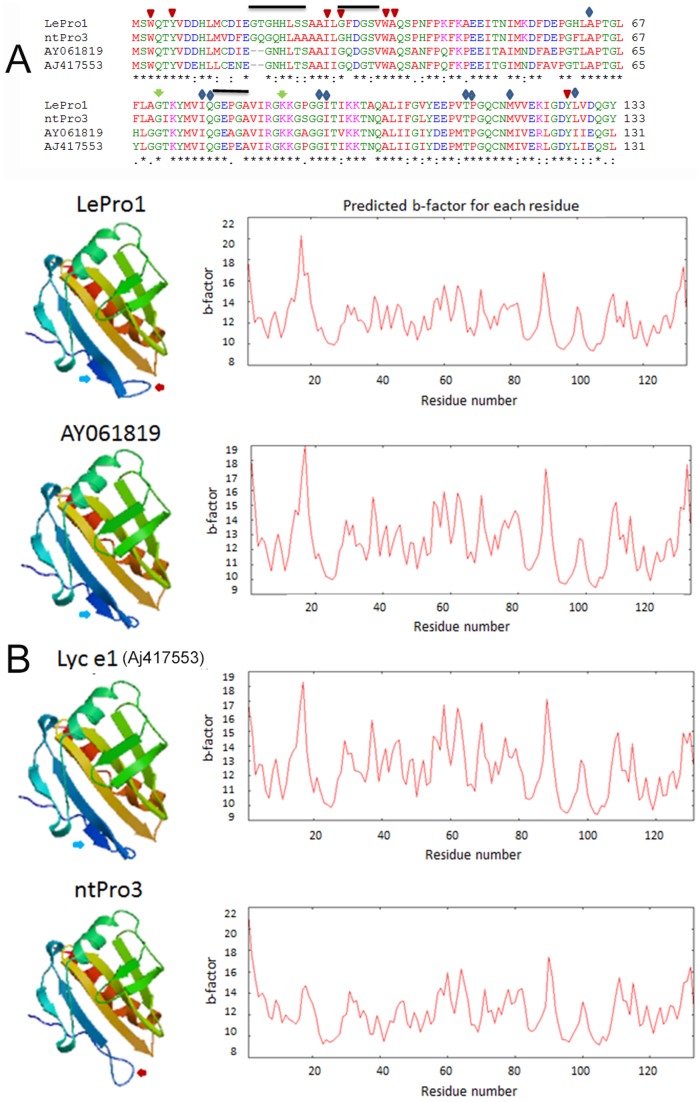
Comparison of protein sequences and 3-D structures of three tomato profilins and a tobacco pollen profilin. **A**, amino acid sequence alignment of three tomato profilins with tobacco pollen profilin, ntPro3. The consensus sequence is indicated by star “*”, non-consensus sequence by colon “:” or dot “.” and missing sequence is indicated by “**-**”. The sequence data were obtained from Genbank with accession numbers of U50195 for tomato pollen profilin *LePro1*, AJ417553 for tomato fruit profilin Lyc e1, AY061819 for another tomato fruit profilin, and X93466 for tobacco pollen profilin ntPro3. **B**, 3-D protein structures were analyzed using Swiss Model (www.swissmodel.expasy.org). Ribbon structures of helices and β sheets are shown on the left panels. Additional loop structures are present in two pollen profilins, *LePro1* and ntPro1 (Red arrow). Additional strand for β sheet are present in the tomato profilins (Blue arrow). The plots of the predicted B-factor for each residue are present on the right panel.

To compare the sequence similarity of *LePro1* with other plant profilins, we carried out a blast search in the NCBI database using the deduced amino acid sequence of *LePro1* as a query. We found that *LePro1* shared different levels of sequence similarity with at least 100 profilin accessions in dicot plants. Phylogenic analysis revealed 4 major clusters (Fig. S1). Cluster 1 contains profilins mainly from Medicago, Populus, tomato fruit and potato tuber. Cluster 2 contains profilins from various species including lotus, cotton, grape, cucumber and hazelnut. Cluster 3 contains 50 profilin accessions from olive and one accession from lily. *LePro1* was clustered into cluster 4, together with its genomic sequence and four tobacco profilins. A partial sequence for profilin in petunia x hybrid is also classified in this cluster (Fig. S1). Of those, the tobacco pollen profilin, ntPro3 [33] showed 87% sequence similarity with *LePro1*. Interestingly, three accessions of non-pollen tomato profilins showed lower sequence similarities with *LePro1* and were clustered into cluster 1. Of those, two tomato accessions, AY061819 and AJ417553 (Lyc e1) were isolated from tomato fruits by RT-PCR using a set of primers derived from *LePro1* [34]. Sequence alignment indicated that the two fruit profilins shared 76–78% of similarity with *LePro1* and were two amino acids shorter than *LePro1* and ntPro3. Residues Gly19 and His20 were missing in the fruit profilins (Fig. 11A). Sequence comparison also revealed that the basic binding sites such as poly-L-proline binding sites (Fig. 11A, triangle), PIP2 binding sites (Fig. 11A, arrow) and actin binding sites (Fig. 11A, diamond) are conserved among the four profilins. Sequence comparison revealed significant variability in post-translational motifs such as [^17^G(T/Q)G(H/Q/N)HL(S/A/S)(S/A)^22^], [^27^G(F/H/Q)DG(S/T)^31^], [^79^GE(P/A)(G/E)^82^] (Fig. 11A, black bar) and phosphorylation sites (Fig. 11A, S/T/Y residues). Characterization of pollen profilins including a large number of olive profilins reveals multifunctionality regulated by post-translational modifications including phosphorylation [22]. Online analysis of 3-D structure was conducted among *LePro1*, ntPro3, and the two tomato fruit profilins using the Swiss Model (www.swissmodel.expasy.org). We found that all four profilins showed the basic folding structure containing a central β sheet sandwiched between the N-terminal helix on one side and two short helices on the other (Fig. 11B). The difference is that an additional strand was found in tomato profilins but not in tobacco profilin (Fig. 11B, blue arrows). Also an additional loop structure was found in the pollen profilins but not in the fruit ones (Fig. 11B, red arrows). Similar plot profiles of the predicted B-factor were found in the three tomato profilins (Fig. 11B, right panel). However, B-factor values were higher in the pollen profilins than those of fruit ones (Fig. 11B, right panel).

## Discussion

### Profilin Isoforms in Tomato Pollen

Profilin multigene families have been reported in a number of species including maize, Arabidopsis, *Glycine max, Olea europaea, Medicago truncatula, Vitis vinifera, Brassica napus* and *Mercurialis annua* [7], [22], [35]. Multiple profilin isoforms were also found in pollen and multifunctionality of isovariants has been investigated [22]. We also attempted to isolate different isoforms in tomato pollen. First, we screened a tomato pollen cDNA library using *LePro1* as a probe. A large number of colonies were screened and only one positive clone was obtained. Sequence analysis revealed that this cDNA clone encodes the same profilin as LePro1 (data not shown). Only one nucleotide substitution occurred in the coding region but that did not change the amino acid sequence. This single nucleotide polymorphism may be due to the genotypic variation as we used a cDNA library from another tomato cultivar VF36, and not Moneymaker. This result was confirmed by the blast search in the tomato full-length cDNA database where only one homologous cDNA was found to encode *LePro1*. Analyzing genomic organization of *LePro1* also showed single copy gene encoding *LePro1*. All these findings pointed out that the tomato pollen profilin is likely encoded by a single gene and no additional copy has been found to be a homolog to *LePro1* so far. This result was not expected as multigene families were found in other plant species. However, it has been reported that a single profilin transcript was found in the root nodules of *Phaseolus vulgaris* and it gave rise to multiple profilin isoforms after transcription [36]. In animals, single copy profilin gene is specifically expressed in most of the coelomocytes of the adult purple sea urchin, *Strongylocentrotus purpuratus* [37]. Previous studies on the biochemistry of tomato pollen by Darnowski [29] indicates that multiple profilin isoforms are present based on the gel-shift assay. The possible explanation is that although profilin in tomato pollen is encoded by a single gene, multiple isoforms may be generated by post-translational modification such as phosphorylation, and in turn displaying multifunctionality in tomato pollen.

### *LePro1* is Encoded by a Pollen-specific Profilin and Divergent from the Fruit Profilins

Our previous study [23] and the gene expression result from the present study indicate that *LePro1* is a pollen-specific profilin. Sequence alignment showed higher similarity between *LePro1* and the tobacco pollen profilin, ntPro3 [33] than that of tomato fruit profilins, suggesting an organ-specificity. Multifunctional isovariants could have risen by post-transcriptional phosphorylation. The analysis of 3-D structure of the four profilins showed similar folding structure (Fig. 11B). However, B-factor values in pollen profilins are higher than those in fruit ones. The B-factor is a value that can be used to indicate the mobility of individual atoms. Higher B-factor value indicates high atomic mobility in the pollen profilins. Another difference between the pollen and fruit profilins is that an insertion of a loop structure at residues 16–20 is present in the pollen profilins but not in the fruit ones (Fig. 11B). To confirm this finding, we extended the 3-D comparison with pollen profilins of Arabidopsis, *Brassica* and maize, and found that they all had similar loop structure at the same region. According to the structure based sequence alignment of 35 profilins of different sources including plants, animals and humans [38], the residues 13–20 are one of the most divergent regions in profilin. This region forms a loop between helix 1 and β strand 1, which is also one of the most distinguished structures for plant profilin [38]. It has been suggested that, in Arabidopsis, two residues in this region, His15 and Thr17, together with Asp115 are involved in stabilizing strand 1 and 7 of the β sheet at the edge of the actin-binding site by form a water-mediated hydrogen bond that is not found in non-plant profilins. Thus, it is likely that the additional loop of protein structure present in *LePro1* and other pollen profilins may have specific roles.

### An Essential Role of LePro1 in Pollen Hydration, Germination and Tube Growth

Using antisense strategy, we have demonstrated successful suppression of the *LePro1* gene expression at both transcription and translation levels in A2 and A3 antisense lines. The suppression of profilin expression could result in less than optimal levels of profilactin complex in pollen. One could speculate that such a reduction, in turn, lead to poor germination and/or tube growth. The in vitro germination results lead credence to such a speculation. Ramachandran et al. [18] analyzed *in vivo* functions of *Arabidopsis* profilin (PFN-1) by generating transgenic plants carrying a 35S-PFN-1 or a 35S-antisense PFN-1 transgene. Their results also showed that under expression of profilin resulted in two-fold reduction in hypocotyl cell length when compared to the wild type. The binding ability of profilins to polyphosphoinositides and proline-rich proteins suggests that profilin may be involved in signaling pathways that affect F-actin organization [17]. Therefore, it is likely that the poor germination and tube growth seen in the A2 and A3 lines maybe due to the down-regulation of endogenous profilin, which may in turn lead to an abnormal actin polymerization and/or actin-cytoskeleton disorganization. Indeed, our F-actin staining results demonstrated a disorganized actin cytoskeleton in the antisense pollen tube (Fig. 10). This assumption is supported by the recent study on the profilin loss-of-function phenotype in *Physcomitrella patens* [21]. In this study, the authors used transient RNA interference approach and demonstrated that F-actin was disorganized and the tip growth was inhibited in profilin-defective moss cells. The tip growth of *Physcomitrella* cell is similar to that of pollen tube [21]. Our F-actin staining and quantification results demonstrate disorganization of F-actin and a lower level in antisense pollen. We therefore suggest that the profilin, *LePro1* in conjunction with perhaps other cytoskeletal proteins, plays a regulatory role in the proper organization of F-actin in tomato pollen tubes through promoting actin assembly.

There is also evidence to suggest that plant profilins are involved in signal transduction cascades [39]. Clarke et al. [40] added a slight excess of native profilin from pollen of *Papaver rhoeas* to cytosolic extracts and found its interaction with the phosphorylation of several proteins, and suggested that, in addition to the regulation of the actin cytoskeletal protein assembly, pollen profilin can regulate protein kinase or phosphatase activity. This indicates that profilin may be involved in signaling pathways which can in turn regulate pollen germination and tube growth. Studies on maize profilin have shown that there are two functionally distinct classes of profilin isoforms [19]. It is thus conceivable that in the pollen of A2 and A3 antisense lines, down-regulation of profilin affects both actin cytoskeleton modulation and signaling pathways. For example, the failure of a significant number of A2 and A3 pollen grains to hydrate could be attributed to disrupted signaling (Fig. 4H–I). However, since the process of pollen germination and tube growth involves many regulatory factors [5], a careful synthesis of all biochemical, molecular and cytological studies from the same species would be required to elucidate the precise *in vivo* roles of the profilin.

## Supporting Information

Figure S1 **BLAST and Phylogenic analysis of plant pollen profilins.** The cDNA sequence of *LePro1* was used as a query for blast search in the nucleotide database of the National Center for Biotechnology Information (NCBI). One hundred accessions of profilin sequences showing high similarity to *LePro1* were clustered using neighbor-joining tree in the same website. The query sequence was highlighted by yellow color.
(TIF)

## References

[1] Parthasarathy MV, Perdue AM, Witzum AW, Alvernaz J (1985) Actin network as a normal component of the cytoskeleton in many vascular plant cells. Am J Bot 72: 1318–1323.

[2] Staiger CJ, Schliwa M (1987) Actin localization and function in higher plants. Protoplasma 141: 1–12.

[3] Seagull RW (1989) The plant cytoskeleton CRC Crit Rev Plant Sci. 8: 131–167.

[4] Aderem A (1992) Signal transduction and the actin cytoskeleton: the roles of MARCKS and profilin. Trends Biochem Sci 17: 438–443. 1455513 10.1016/0968-0004(92)90016-3

[5] Taylor LP, Hepler PK (1997) Pollen germination and tube growth. Annu Rev Plant Physiol Plant Mol Biol 48: 461–491. 15012271 10.1146/annurev.arplant.48.1.461

[6] Stossel TP, Chaponier C, Ezzell RM, Hartwig JH, Janmey PA, et al (1985) Nonmuscle actin binding proteins. Ann Rev Cell Biol 1: 353–402. 3030380 10.1146/annurev.cb.01.110185.002033

[7] Staiger CJ, Goodbody KC, Hussey PJ, Valenta R, Drobak BK, et al (1993) The profilin multigene family of maize: differential expression of three isoforms. Plant J 4: 631–641. 8252067 10.1046/j.1365-313x.1993.04040631.x

[8] Tilney LG, Bonder EM, Coluccio LM, Mooseher MS (1983) Actin from Thyone sperm assembles on only one end of an actin filament, a behavior regulated by profilin. J Cell Biol 97: 112–124. 6863386 10.1083/jcb.97.1.112 PMC2112487

[9] Buss F, Temm-Grove C, Henning S, Jockusch BM (1992) Distribution of profilin in fibroblasts correlates with the presence of highly dynamic actin filaments. Cell Motil Cytoskel 22: 51–61. 10.1002/cm.970220106 1581979

[10] Cooley L, Verheyen E, Ayers K (1992) Chickadee encodes a profilin required for intercellular cytoplasm transport during *Drosophila oogenesis.* . Cell 69: 173–184. 1339308 10.1016/0092-8674(92)90128-y

[11] Grote M, Vrtala S, Valent R (1993) Monitoring of two allergens, Bet v I and profilin, in dry and rehydrated birch pollen by immunogold electron microscopy and immunoblotting. J Histochem Cytochem 41: 745–750. 8468456 10.1177/41.5.8468456

[12] Goldschmidt-Clermont PJ, Machesky LM, Baldassare JJ, Pollard TD (1990) The actin-binding protein profilin binds to PIP2 and inhibits its hydrolysis by phospholipase C. Science. 247: 1575–1578. 10.1126/science.2157283 2157283

[13] Machesky LM, Goldschmidt-Clermont PJ, Pollard TD (1990) The affinities of human platelet and *Acanthamoeba* profilin isoforms for polyphosphoinositides account for their relative abilities to inhibit phospholipase C. Cell Regul. 1: 937–950. 10.1091/mbc.1.12.937 PMC362863 1966040

[14] Vojtek A, Haarer B, Field J, Gerst J, Pollard TD, Brown S, et al (1991) Evidence for a functional link between profilin and CAP in yeast *S. cerevisiae* . Cell 66: 497–505. 1868547 10.1016/0092-8674(81)90013-1

[15] Witke W (2004) The role of profilin complexes in cell motility and other cellular processes. Trends in Cell Biol 14: 461–469. 15308213 10.1016/j.tcb.2004.07.003

[16] Valenta R, Duchene M, Pettenburger K, Sillaber C, Valent P, et al (1991) Identification of profilin as a novel pollen allergen; IgE autoreactivity in sensitized individuals. Science 253: 557–559. 1857985 10.1126/science.1857985

[17] Gibbon BC, Staiger CJ (2000) Profilin. In Staiger CJ, Baluska F, Volkmann D, Barlow P, eds. Actin, A dynamic Framework for Multiple Plant Functions. Dorgrecht, The Netherlands, Kluwer Academic Publishers. 45–65.

[18] Ramachandran S, Christensen HEM, Ishimaru Y, Dong C–H, Chao-Ming W, et al (2000) Profilin plays a role in cell elongation, cell shape maintenance, and flowering in Arabidopsis. Plant Physiol. 124: 1637–1647. 10.1104/pp.124.4.1637 PMC59862 11115881

[19] Kovar DR, Dr∅bak BK, Staiger CJ (2000) Maize profilin isoforms are functionally distinct. Plant Cell 12: 583–98. 10760246 10.1105/tpc.12.4.583 PMC139855

[20] Kandasamy MK, McKinney EC, Meagher RB (2002) Plant profilin isovariants are distinctly regulated in vegetative and reproductive tissues. Cell Motility Cytoskel 52: 22–32. 11977080 10.1002/cm.10029

[21] Vidali L, Augustine RC, Kleinman KP, Bezanilla M (2007) Profilin is essential for tip growth in the moss *Physcomitrella patens.* . Plant Cell 19: 3705–3722. 17981997 10.1105/tpc.107.053413 PMC2174871

[22] Jimenez-Lopez JC, Morales S, Castro AJ, Volkmann D, Rodríguez-García MI, et al (2012) Characterization of profilin polymorphism in pollen with a focus on multifunctionality. PLoS ONE 7(2): e30878 doi:10.1371/journal.pone.0030878 22348028 10.1371/journal.pone.0030878 PMC3279341

[23] Yu L–X, Nasrallah J, Valenta R, Parthasarathy MV (1998) Molecular cloning and mRNA localization of tomato pollen profilin. Plant Mol Biol 36: 699–707. 9526502 10.1023/a:1005971327353

[24] Fulton TM, Chunwongse J, Tanksley SD (1995) Microprep protocol for extraction of DNA from tomato and other herbaceous plants. Plant Mol Biol Rep 13: 207–209.

[25] Sambrook J, Fritsch EF, Maniatis T (1989) Molecular cloning, a laboratory manual 2nd edition Cold Spring Harbor Lab Press.

[26] Fillatti JJ, Kiser J, Rose B, Comai L (1987) Efficient transformation of tomato and the introduction and expression of a gene for herbicide tolerance. In D J Nevins and R A Jones. Eds. Plant Biology, Tomato biotechnology 4: 199–210.

[27] Frary A, Earle ED (1996) An examination of factors affecting the efficiency of *Agrobacterium*-mediated transformation of tomato. Plant Cell Rep 16: 235–240. 24177560 10.1007/BF01890875

[28] Li F, Dey M, He C, SangwanV, Wu X, et al (2003) Rapid PCR-based determination of transgene copy number in rice. Plant Mol Biol Rep 21: 73–80.

[29] Darnowski DW (1997) Biochemistry and immunolocalization of profilin in tomato pollen PhD Thesis Cornell University Ithaca, NY.

[30] Andersland JM, Fisher D, Wymer CL, Cyr RJ, Parthasarathy MV (1994) Characterization of monoclonal antibodies raised against plant actin. Cell Motil and Cytoskel 29: 339–344. 10.1002/cm.970290406 7859296

[31] Bollag DM, Edelstein SJ (1991) Protein methods. A John Wiley & Son, Inc Publication, New York.

[32] Gibbon BC, Kovar DR, Staiger CJ (1999) Latrunculin B has different effects on pollen germination and tube growth. Plant Cell 11: 2349–2363. 10590163 10.1105/tpc.11.12.2349 PMC144132

[33] Mittermann I, Heiss S, Kraft D, Valenta R, Heberle-Bors E (1996) Molecular characterization of profilin isoforms from tobacco (*Nicotiana tabacum*) pollen. Sex Plant Reprod 9: 133–139.

[34] Westphal S, Kolarich D, Foetisch K, Lauer I, Altmann F, et al (2003) Molecular characterization and allergenic activity of Lyc e 2 (beta -fructofuranosidase), a glycosylated allergen of tomato. European J Biochem 270: 1327–1337. 12631291 10.1046/j.1432-1033.2003.03503.x

[35] Huang S, McDowell JM, Weise MJ, Meagher RB (1996) The Arabidopsis profilin gene family, evidence for an ancient split between constitutive and pollen-specific profilin genes. Plant Physiol 111: 115–126. 8685262 10.1104/pp.111.1.115 PMC157818

[36] Guillen G, Valdes-Lopez V, Noguez R, et al (1999) Profilin in *Phaseolus vulgaris* is encoded by two genes (only one expressed in root nodules) but multiple isoforms are generated *in vivo* by phosphorylation on tyrosine residues. Plant Cell 19: 497–508. 10.1046/j.1365-313x.1999.00542.x 10504572

[37] Smith LC, Britten RJ, Davison EH (1992) SpCoell: a sea urchin profilin gene expressed specifically in coelomocytes in response to injury. Mol Biol of the Cell 3: 403–414. 10.1091/mbc.3.4.403 PMC275591 1498361

[38] Thorn KS, Christensen HE, Shigeta R, Huddler D, Shalaby L, et al (1997) The crystal structure of a major allergen from plants. Structure 5: 19–32. 9016723 10.1016/s0969-2126(97)00163-9

[39] Dr∅bak BK, Watkins PAC, Valenta R, Dove SK, Lloyd CW, et al (1994) Inhibition of plant plasma membrane phosphoinositide phospholipase C by the actin-binding protein, profilin. Plant J 6: 389–400.

[40] Clarke SR, Staiger CJ, Gibbon BC, Franklin-Tong VE (1998) A potential signaling role for profilin in pollen of *Papaver rhoeas.* . Plant Cell 10: 967–979. 9634585 10.1105/tpc.10.6.967 PMC144038

