# Supplementary figures and images for: Molecular and Cellular Characterization of the Tomato Pollen Profilin, LePro1
Source: PLoS One. 2014 Jan 21;9(1):e86505. doi: 10.1371/journal.pone.0086505 (PMC3897733; doi:10.1371/journal.pone.0086505)

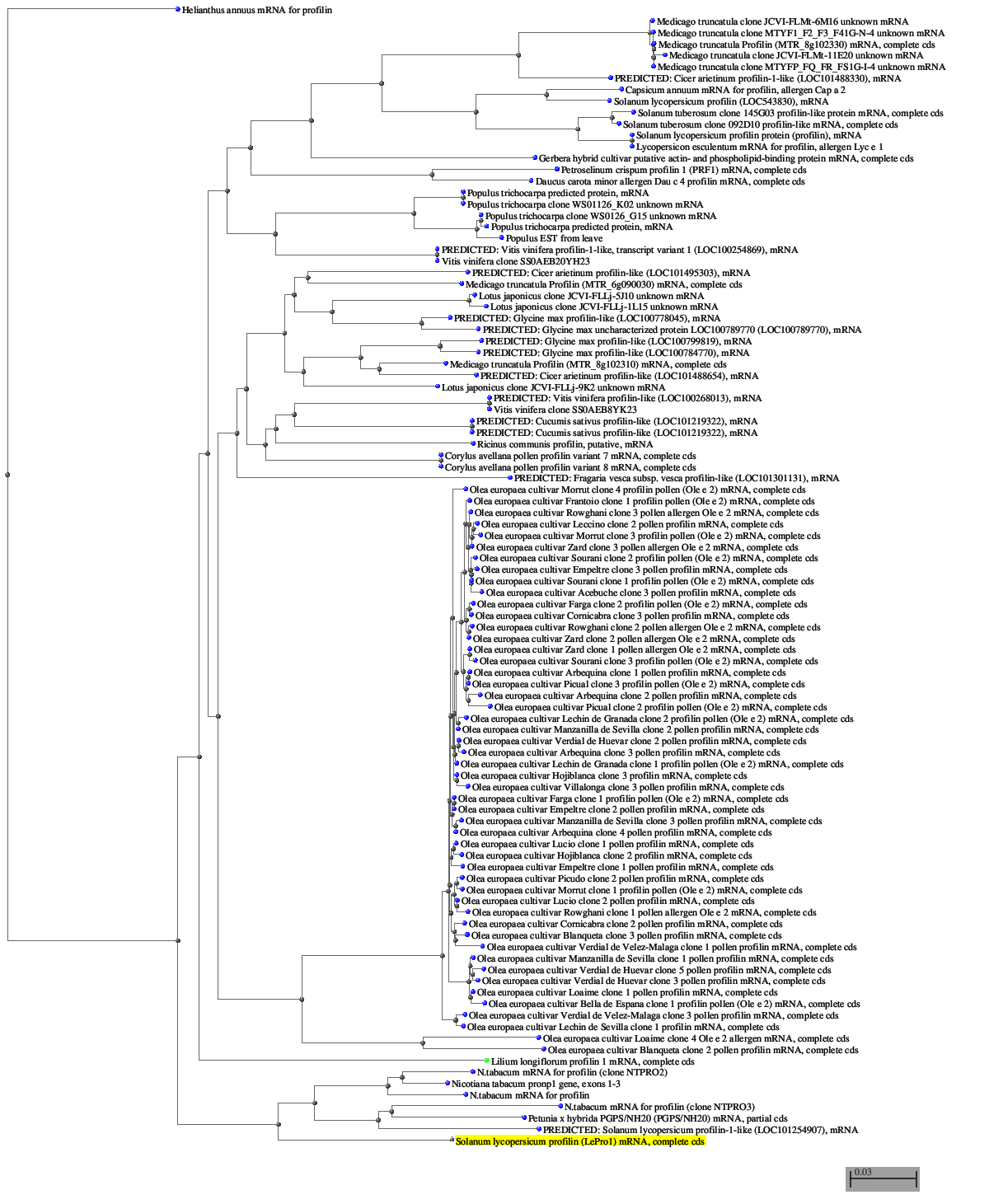

Supplement: Figure S1 — BLAST and Phylogenic analysis of plant pollen profilins. The cDNA sequence of LePro1 was used as a query for blast search in the nucleotide database of the National Center for Biotechnology Information (NCBI). One hundred accessions of profilin sequences showing high similarity to LePro1 were clustered using neighbor-joining tree in the same website. The query sequence was highlighted by yellow color. (TIF) [file pone.0086505.s001.tif]
